# Supplementary material for: Population Parameters and Growth of Riptortus pedestris (Fabricius) (Hemiptera: Alydidae) under Fluctuating Temperature
Source: Insects. 2022 Jan 21;13(2):113. doi: 10.3390/insects13020113 (PMC8876695; doi:10.3390/insects13020113)
Supplement: Supplementary file 1 [file insects-13-00113-s001.zip › insects-1538120-SI.pdf]

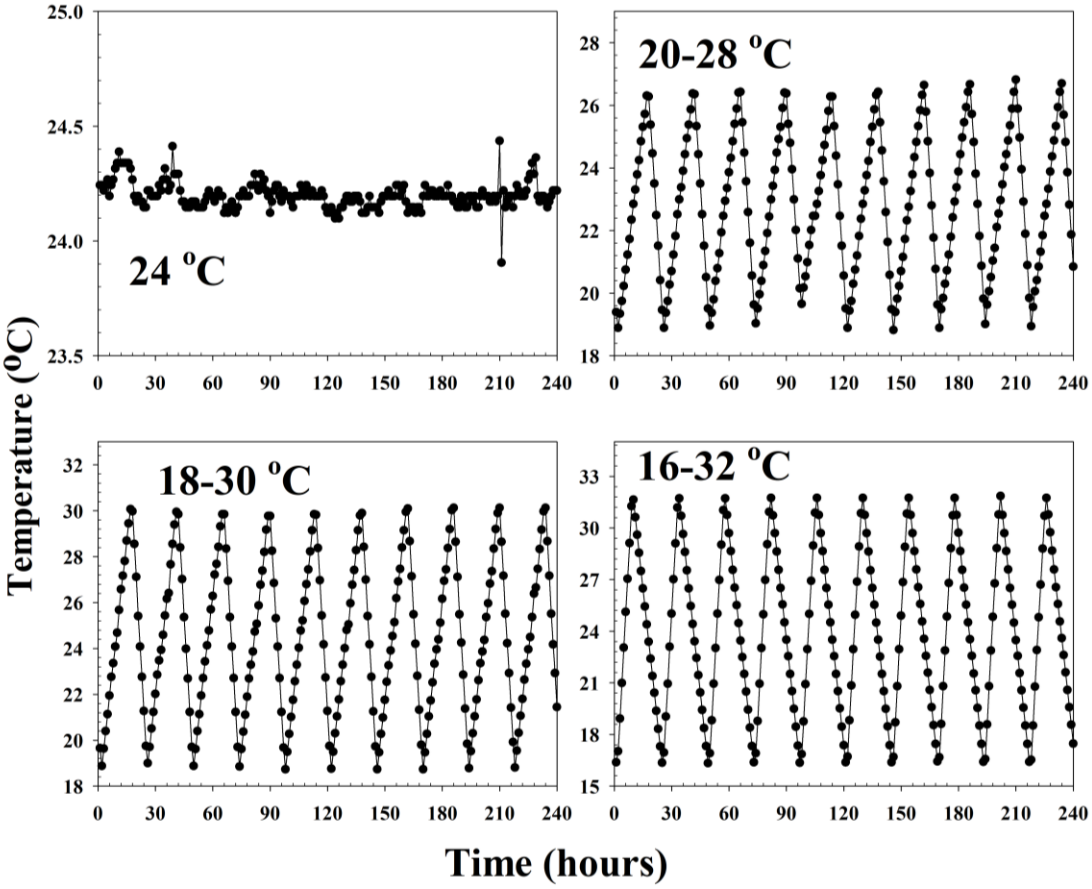

Figure S1. Stepwise process used in temperature cabinets for constant and fluctuating temperature regimes.
